# The Effectiveness of Virtual Reality in Controlling Pain and Anxiety Levels in Four-to-Six-Year-Old Children During Dental Treatment

**DOI:** 10.7759/cureus.51099

**Published:** 2023-12-26

**Authors:** Omar S Almajed, Rakan Alhujhuj, Eyad Alshaheen, Abdullatif Almujhim, Mohammed Albutayh, Ravi G Raghunath, Gauri Lele

**Affiliations:** 1 Pediatric Dentistry, Imam Abdulrahman Bin Faisal University, Dammam, SAU; 2 Dental Public Health, King’s College London, London, GBR; 3 Dental Department, King Abdulaziz National Guard Hospital, Al Hofuf, SAU; 4 Dentistry, Private Practice, Riyadh, SAU; 5 Dentistry, Private Practice, Al Ahsa, SAU; 6 Dentistry, Private Practice, Al Khobar, SAU; 7 Department of Preventive Dental Sciences, King Faisal University, Al Ahsa, SAU; 8 Pediatric and Preventive Dentistry, Navrachana University, Gujarat, IND

**Keywords:** distraction techniques, pain management, pediatric dentistry, dental anxiety, virtual reality

## Abstract

Background and objective

Oral health is critical for children's overall well-being; however, dental anxiety often leads to the avoidance of necessary dental care among children. Virtual reality (VR) has emerged as a successful adjunctive tool in various clinical settings, including pain management. This cohort study explores the use of VR technology in reducing anxiety and pain perception during dental procedures for children. The primary objective of this study was to assess the efficacy of VR distraction in managing pain and anxiety levels in children aged four to six years during dental procedures. Additionally, the study aimed to compare children's anxiety levels with and without VR eyeglasses.

Methodology

This single-blind, crossover clinical trial was conducted at the Dental Complex of King Faisal University, Al Ahsa, Kingdom of Saudi Arabia. Of the 200 children screened initially, 20 aged four to six years who met the inclusion criteria were selected, and ethical approval for the study was obtained. The study comprised two groups: a control group and an experimental group. Dental procedures were conducted both with and without the use of VR, employing a split-mouth design.

Results

Our findings provide important insights into the clinical impact of using VR technology to alleviate children's anxiety during dental clinic visits. In our study, we used ANOVA and Tukey's post hoc test to evaluate the effect of VR glasses on vital metrics in children during dental procedures. Our results showed no significant differences before and after using the VR glasses, indicating limited effectiveness in reducing anxiety in this context.

Conclusion

Based on our findings, we reject the assumption that VR devices are highly effective in diverting children's attention and reducing their anxiety and pain during dental procedures; we recommend further investigations to identify potential confounding factors that could modulate the utility of VR in medical settings.

## Introduction

Oral health constitutes a critical aspect of overall well-being in children, and dental procedures form a common part of maintaining oral health. However, these procedures can often be associated with pain and anxiety, particularly in pediatric patients. Managing these challenges is crucial for ensuring not only the effectiveness of the dental treatment but also for maintaining the psychological well-being of the child. In recent years, virtual reality (VR) has emerged as a promising tool in this regard. The impact of VR on pain and anxiety management in pediatric dentistry has been the subject of various studies. VR distraction has been shown to significantly reduce pain perception and state of anxiety in children aged five to eight years during short invasive dental procedures [1]. Additionally, VR eyeglasses have been found to significantly improve child behavior, pain perception, and anxiety scores during dental treatment [2]. This indicates that VR can be an effective distraction tool to ease pain and anxiety in children receiving painful dental procedures [3].

The use of VR in pediatric dentistry not only decreases pain perception and anxiety scores during dental treatment but also enhances the overall experience of the child undergoing these procedures [4]. This is particularly important as dental anxiety can lead to avoidance of necessary dental care, which in turn can have long-term adverse effects on oral health. VR distraction techniques have been effective in reducing pain perception and anxiety levels in children undergoing restorative dental treatment, making the procedures more tolerable and less traumatic [5]. Furthermore, the use of VR has been shown to significantly reduce pain and anxiety during local anesthesia in children receiving dental treatment [6]. This is crucial as the administration of local anesthesia is often one of the most anxiety-inducing aspects of dental treatment for children. The significant impact of VR in reducing procedural pain and anxiety in children, especially in dental studies, underscores its potential as a valuable tool in pediatric dentistry [7].

To sum up, the integration of VR into pediatric dental practices offers a novel and effective approach to managing the challenges of pain and anxiety in young patients. Its ability to distract and relax children during dental procedures not only improves their immediate experience but also potentially helps in building a more positive attitude towards oral healthcare in the long term. Hence, this study aims to evaluate the effectiveness of VR in reducing pain and anxiety in pediatric dental patients. It will focus on assessing how VR as a distraction tool impacts children's experience during dental procedures, potentially influencing their long-term attitude toward oral healthcare. This research seeks to provide insights for enhancing child-friendly dental care practices.

## Materials and methods

We conducted a single-blind, crossover clinical trial at the Dental Complex of King Faisal University, Al Ahsa, Kingdom of Saudi Arabia, after obtaining ethical approval from the University's Research Ethics Committee. An educational program was carried out in preschools to screen and include children in this study. After obtaining written permission from preschool authorities and parents, 200 children from five preschools in Al Hofuf, Al Mubarraz, and other areas of Al Ahsa were screened.

The inclusion criteria for this study were specific and as follows: children aged four to six years with no past dental experience, a behavior rating of 3 or 4 on Frankl's Behavior Rating Scale (FBRS), the presence of initial to moderate bilateral class 1 carious lesions in primary molars, and the willingness of both child and parents to participate in the study. Conversely, children were excluded if they were medically compromised or had special healthcare needs, had a behavior rating of 1 or 2 on FBRS, presented with any dental emergency, or had prior dental experience. From the screened group, 20 children meeting these criteria were selected.

In our study, informed consent was obtained from the parents of 20 children who met our selection criteria. We employed a split-mouth design, dividing 40 teeth into two groups: Group I (control) and Group II (experimental). Five coinvestigators treated eight teeth each (four control and four experimental) over three visits. The children underwent three dental visits at two-week intervals, involving prophylaxis and two dental restoration visits. Bilateral molar teeth were randomly assigned to either the control or experimental group. The first visit included a comprehensive examination and oral prophylaxis. During the second visit, class I cavity preparation and restoration were performed, without VR for Group I and with VR for Group II. The third visit involved treating the other group with the alternate method. All cavity preparations and restorations were similar, using MEDIFIL GICs for restoration by Promedica (Promedica Dental Material GmbH, Neumünster, Germany).

To assess anxiety levels and reactions toward the dental appointment, children were asked to choose from eight pictures from the Venham Picture test (VPT) at the beginning of each treatment. The Sound-Eye-Motor (SEM) scale was used to assess children's behavior before, during, and after each procedure, recorded by a third observer through observations of the child's sound, eye, and motor reactions. Additionally, a pulse oximeter was used to record oxygen saturation and pulse rate changes.

For the VR visits, children were educated about the VR device and they chose to watch an episode of "Tom & Jerry" or "SpongeBob" during the treatment. The VR device was fitted to the child's eyes, and the procedure was started, with all scales obtained as in previous appointments. After the procedure, children were asked to sit for five minutes to avoid visual disturbances. All operators in the study were of the same academic level and class, having undergone similar clinical training and possessing comparable clinical expertise.

**Figure 1:**
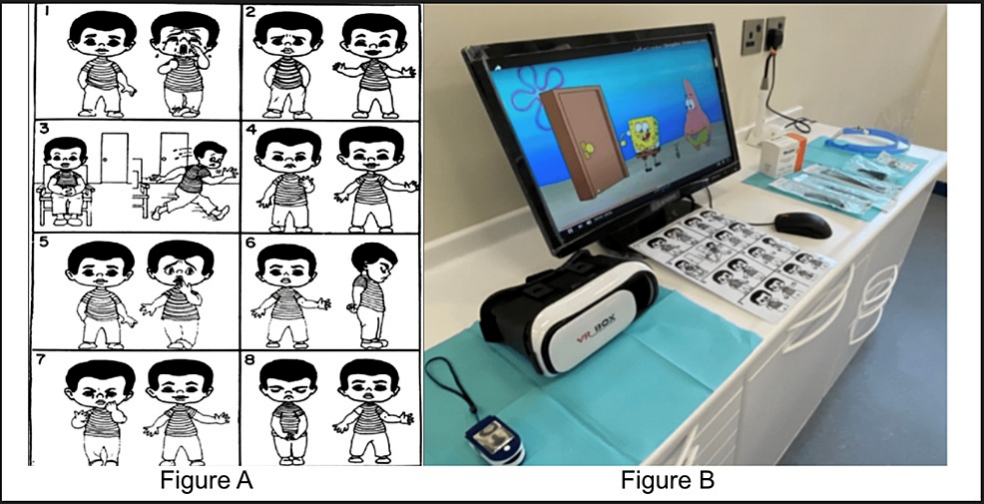
Venham Picture test, pulse oximeter, and VR device Panel A depicts Venham's Picture test with eight illustrated sequences of a character expressing different emotions and performing various actions. Panel B shows a pulse oximeter, a VR headset, and a computer displaying SpongeBob cartoon show VR: virtual reality

## Results

The study was kicked off by assessing SEM scores in a cohort of 20 children during their first (prophylactic), second (dental restoration one), and third (dental restoration two) visits to the dental clinic. The SEM scores, which are rated on a scale ranging from 0 to 10, were documented for each child. The results of the study, as tested by ANOVA and Tukey’s post hoc tests, indicate that there was no statistically significant difference in mean SEM scores among the whole group of children during their prophylaxis visit, the control group (without VR) and the experimental group (with VR) during restoration visits one and two, all of which occurred before any dental interventions were administered (F=1.955, p>0.05). This is detailed in Table 1, with further pairwise comparisons provided in Table 2.

**Table 1:** Comparison of mean SEM scores at different time intervals among all the groups by using the ANOVA test ANOVA: analysis of variance; SEM: Sound-Eye-Motor

Group	N	Mean	Std. deviation	F-value	P-value
First visit	20	3.35	0.671	1.955	0.151
Control group	20	3.10	0.308		
Experimental group	20	3.10	0.308		
Total	60	3.18	0.469		

**Table 2:** Tukey’s post hoc analysis

	First visit	Control group	Experimental group
First visit	-	0.209	0.209
Control group	0.209	-	1.000
Experimental group	0.209	1.000	-

**Figure 2:**
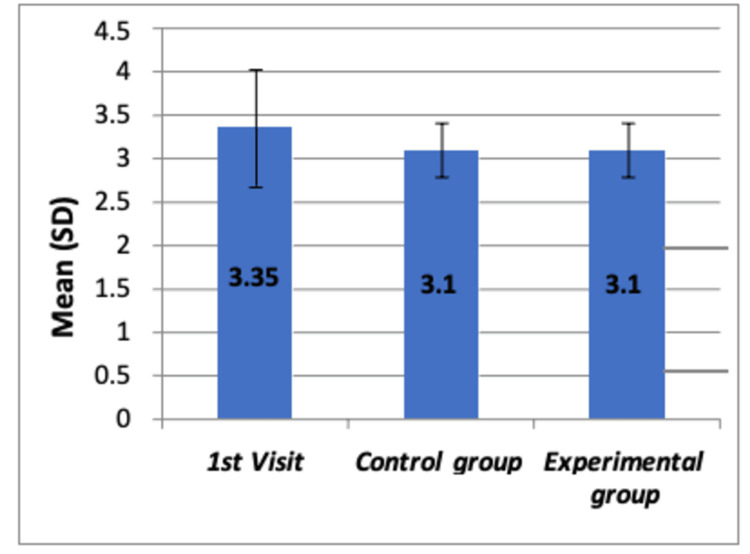
Comparison of mean SEM scores before any dental procedure among all the groups by using Boxplot chart The chart shows no significant differences in mean SEM scores among the three groups (p=0.151) SD: standard deviation; SEM: Sound-Eye-Motor

**Figure 3:**
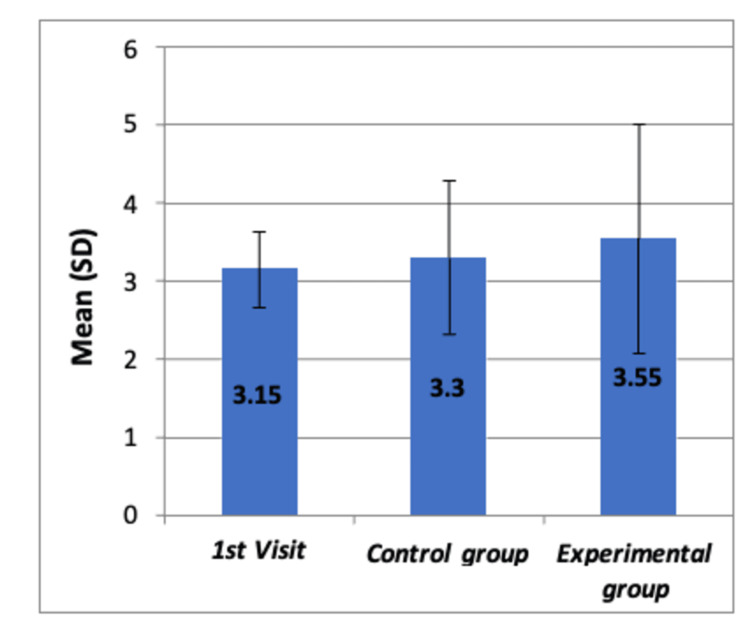
Comparison of mean SEM scores after the dental procedure among all the groups by using Boxplot chart The chart shows no significant differences in mean SEM scores among the three groups (p=0.486) SD: standard deviation; SEM: Sound-Eye-Motor

The same groups' SEM scores were reassessed post-treatment. The ANOVA findings showed no statistically significant difference in mean SEM scores (F=0.731, p>0.05), as shown in Table 3, with pairwise comparisons reported in Table 4. Figures 2-3 illustrate the comparison of SEM scores before and after dental procedures, respectively, with no significant differences in means observed among the groups.

**Table 3:** Post-procedural SEM scores comparison among groups SEM: Sound-Eye-Motor

Group	N	Mean	Std. deviation	F-value	P-value
First visit	20	3.15	0.489	0.731	0.486
Control group	20	3.30	0.979		
Experimental group	20	3.55	1.468		
Total	60	3.33	1.052		

**Table 4:** Tukey’s post hoc analysis

	First visit	Control group	Experimental group
First visit	-	0.895	0.460
Control group	0.895	-	0.736
Experimental group	0.460	0.736	-

**Figure 4:**
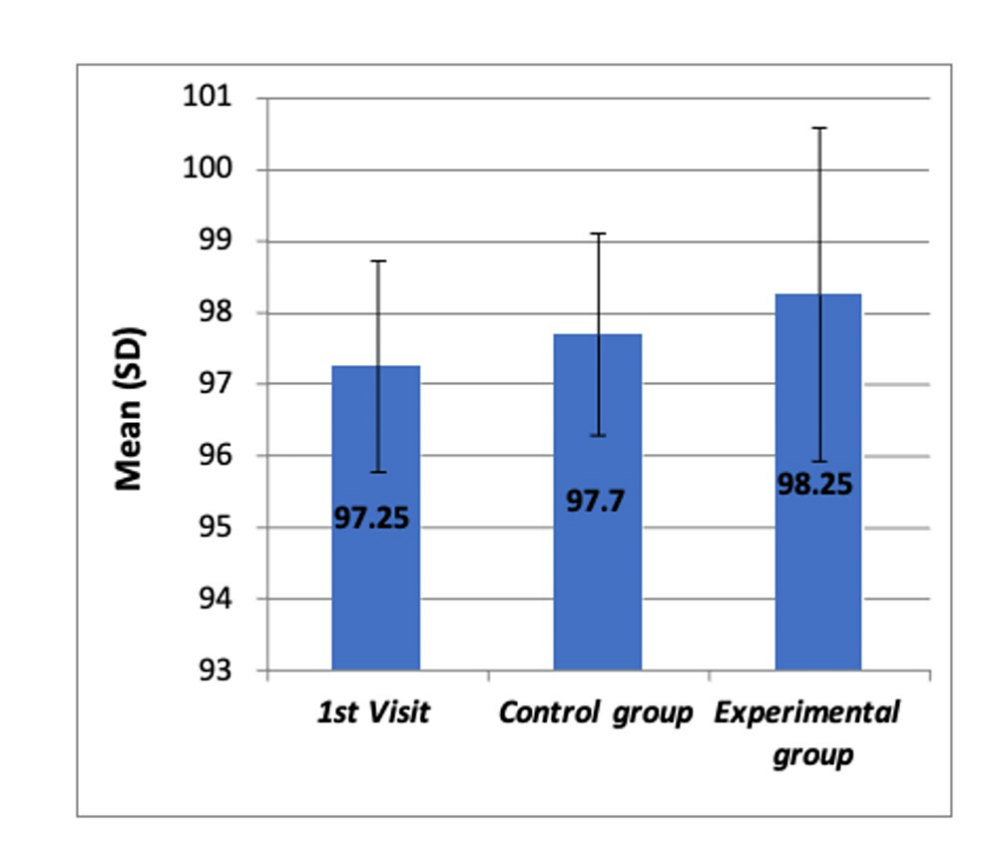
Comparison of mean oxygen saturation levels (SpO2) before any dental procedure among all the groups by using Boxplot chart The chart shows no significant differences in means of oxygen saturation levels (SpO_2_) among the three groups SD: standard deviation

Before starting the dental procedure, the children's oxygen saturation levels (SpO_2_) were assessed. ANOVA results indicated no significant differences between the groups (F=1.557, p>0.05), as seen in Table 5, with Tukey’s post-hoc comparisons in Table 6. The lack of significant difference is also visually presented in Figure 4.

**Table 5:** Comparison of mean oxygen saturation percentages at different time intervals among all groups by using the ANOVA test ANOVA: analysis of variance

Group	N	Mean	Std. deviation	F-value	P-value
First visit	20	97.25	1.482	1.557	0.220
Control group	20	97.70	1.418		
Experimental group	20	98.25	2.337		
Total	60	97.73	1.812		

**Table 6:** Tukey's post hoc analysis

	First visit	Control group	Experimental group
First visit	-	0.709	0.192
Control group	0.709	-	0.599
Experimental group	0.192	0.599	-

**Figure 5:**
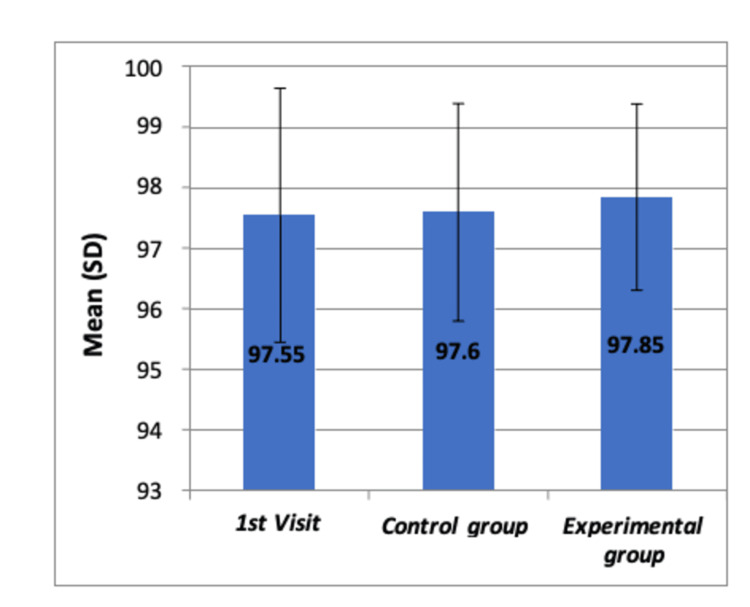
Comparison of mean oxygen saturation levels (SpO2) after the dental procedure among all the groups by using Boxplot chart The chart shows no significant differences in means of oxygen saturation levels (SpO_2_) among the three groups SD: standard deviation

Following the procedures, the SpO_2_ levels were measured again, and no significant changes were observed (F=0.156, p>0.05), as detailed in Table 7, with pairwise comparisons in Table 8. Figure 5 shows the comparison of SpO_2_ levels after dental procedures, confirming no significant differences.

**Table 7:** Oxygen saturation levels after dental procedures across groups

Group	N	Mean	Std. deviation	F-value	P-value
First visit	20	97.55	2.089	0.156	0.856
Control group	20	97.60	1.789		
Experimental group	20	97.85	1.531		
Total	60	97.67	1.791		

**Table 8:** Tukey’s post hoc analysis

	First visit	Control group	Experimental group
First visit	-	0.996	0.861
Control group	0.996	-	0.901
Experimental group	0.861	0.901	-

**Figure 6:**
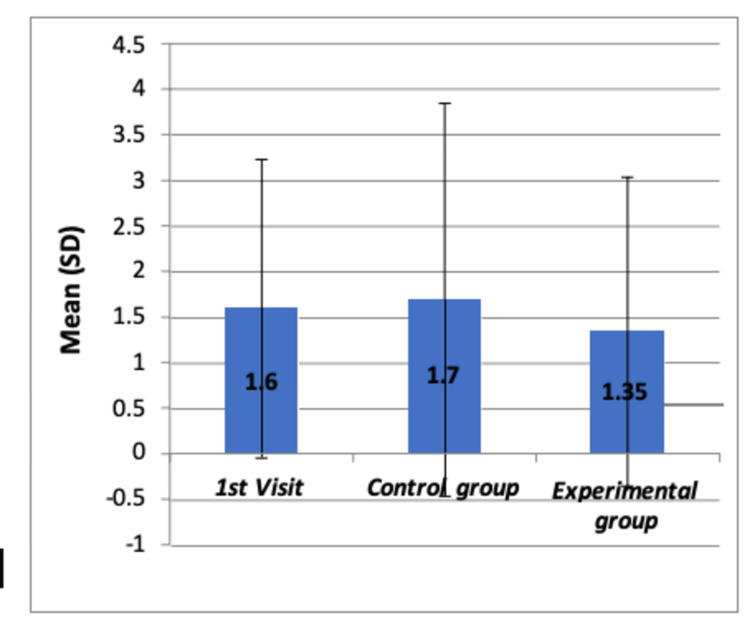
Comparison of mean VPT scores at different time intervals of each dental visit among all the groups by using Boxplot chart The chart shows no significant differences in mean VPT scores among the three groups SD: standard deviation; VPT: Venham Picture test

**Figure 7:**
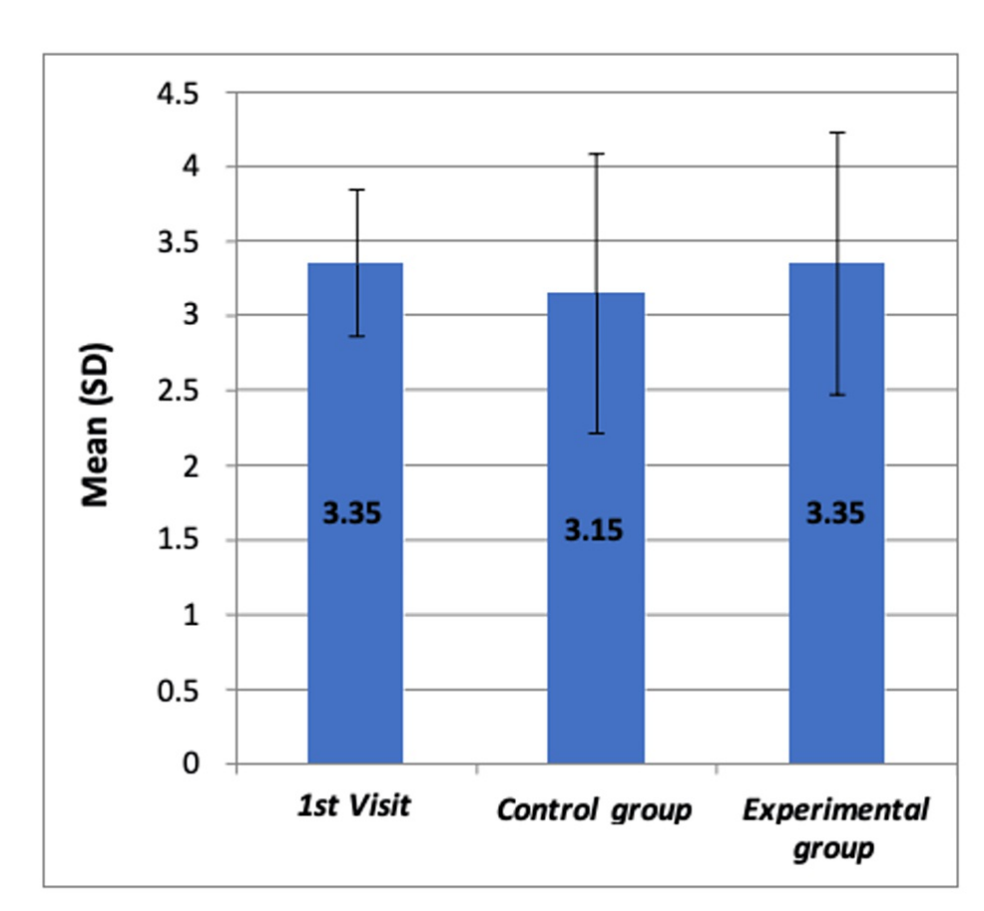
Comparison of mean FBRS at different time intervals of each dental visit among all the groups by using Boxplot chart The chart shows no significant differences in means of FBRS among the three groups FBRS: Frankl's Behavior Rating Scale; SD: standard deviation

The children's behavior was quantitatively assessed using VPT scores and FBRS. The Kruskal-Wallis test found no significant differences in VPT scores (χ2=0.381, p>0.05), which is reported in Table 9, with individual comparisons in Table 10. Figures 6-7 depict the comparison of VPT and FBRS, respectively, with no significant differences noted among the groups.

**Table 9:** Comparison of mean VPT scores among all the groups by using the Kruskal-Wallis test VPT: Venham Picture test

Group	N	Mean	Std. deviation	Chi-square value	P-value
First visit	20	1.60	1.635	0.381	0.826
Control group	20	1.70	2.155		
Experimental group	20	1.35	1.694		
Total	60	1.55	1.817		

**Table 10:** Individual comparison using the Mann-Whitney U test

	First visit	Control group	Experimental group
First visit	-	0.862	0.565
Control group	0.862	-	0.738
Experimental group	0.565	0.738	-

The study also evaluated the FBRS in the same groups. The ANOVA results showed no statistically significant difference in mean FBRS among all children during their prophylaxis visit, non-VR users (control group), and VR users (experimental group) during restoration visits one and two (F=0.426, p>0.05), as documented in Table 11. The Tukey’s post hoc analysis for these scores is detailed in Table 12. The graphical representation of the FBRS at different time intervals of each dental visit among all groups, showing no significant differences, is provided in Figure 7.

**Table 11:** Comparison of mean FBRS among all the groups by using the ANOVA test ANOVA: analysis of variance; FBRS: Frankl's Behavior Rating Scale

Group	N	Mean	Std. deviation	F-value	P-value
First visit	20	3.35	0.489	0.426	0.655
Control group	20	3.15	0.933		
Experimental group	20	3.35	0.875		
Total	60	3.28	0.783		

**Table 12:** Tukey’s post hoc analysis

	First visit	Control group	Experimental group
First visit	-	0.705	1.000
Control group	0.705	-	0.705
Experimental group	1.000	0.705	-

During the initial visit of children to the dental care units, SEM scores, pulse rate, and oxygen saturation values were recorded both before and after the dental procedure for all children. A paired t-test was employed to compare the means of all these parametric measures before and after the intervention. The results revealed that there were no statistically significant differences in mean values before and after dental treatment (p>0.05), as shown in Table 13. Descriptive statistics for the VPT and FBRS for this visit are compiled in Table 14.

**Table 13:** Initial visit behavioral scores for all children (N=20) SD: standard deviation

Variable	Mean ± SD
VPT score	1.60 ± 1.635
FBRS	3.35 ± 0.489

**Table 14:** Initial visit - comparative analysis of SEM, pulse rate, and oxygen saturation pre and post dental examination SEM: Sound-Eye-Motor

Variable	Time interval	N	Mean	Std. deviation	t-value	P-value
SEM	Before	20	3.35	0.671	1.710	0.104
	After	20	3.15	0.489		
Pulse rate	Before	20	89.40	11.686	1.884	0.075
	After	20	84.80	10.556		
Oxygen saturation	Before	20	97.25	1.482	0.623	0.541
	After	20	97.55	2.089		

In the control group (children who did not use VR devices during the dental procedure), SEM scores, pulse rate, and oxygen saturation values were recorded before and after the dental procedure. A paired t-test was used to compare the means of all these parameters before and after the intervention. The results, showing no statistically significant difference (p>0.05), are detailed in Table 15. The descriptive statistics for the VPT and FBRS in the control group are presented in Table 16.

**Table 15:** Comparison of mean SEM scores, pulse rate, and oxygen saturation values at different time intervals in the control group by using paired t-test SEM: Sound-Eye-Motor

Variable	Time interval	N	Mean	Std. deviation	t-value	P-value
SEM	Before	20	3.10	0.308	0.847	0.408
	After	20	3.30	0.979		
Pulse rate	Before	20	85.40	13.040	1.590	0.128
	After	20	88.75	13.924		
Oxygen saturation	Before	20	97.70	1.418	0.248	0.807
	After	20	97.60	1.789		

**Table 16:** Descriptive statistics for VPT and FBRS in the control group (N=20) FBRS: Frankl's Behavior Rating Scale; SD: standard deviation; VPT: Venham Picture test

Variable	Mean ± SD
VPT score	1.70 ± 2.155
FBRS	3.15 ± 0.933

In the experimental group (children who used VR devices during the medical intervention), SEM scores, pulse rate, and SpO_2_ values were again recorded before and after the intervention. The paired t-test results showed no statistically significant differences when compared with the means before dental treatment (p>0.05), as seen in Table 17. The descriptive statistics for the VPT and FBRS in the experimental group, which reflect the behavioral responses to the dental procedure, are presented in Table 18.

**Table 17:** Comparison of mean SEM scores, pulse rate, and oxygen saturation values at different time intervals in the experimental group by using paired t-test SEM: Sound-Eye-Motor

Variable	Time interval	N	Mean	Std. deviation	t-value	P-value
SEM	Before	20	3.10	0.308	1.528	0.143
	After	20	3.55	1.468		
Pulse rate	Before	20	86.95	13.241	1.363	0.189
	After	20	82.60	11.914		
Oxygen saturation	Before	20	98.25	2.337	0.914	0.372
	After	20	97.85	1.531		

**Table 18:** Behavioral assessment scores for the experimental group (N=20) FBRS: Frankl's Behavior Rating Scale; SD: standard deviation; VPT: Venham Picture test

Variable	Mean ± SD
VPT score	1.35 ± 1.694
FBRS	3.35 ± 0.875

## Discussion

Oral hygiene constitutes a pivotal determinant of children's overall health, significantly influenced by the typical developmental process of the child. Research findings have underscored the relationship between normal developmental processes, oral health, and primary tooth eruption in children [8]. The concept of oral health-related quality of life (OHRQOL) is a multidimensional parameter affected by various factors, including dental anxiety, the socioeconomic status of parents, and oral health-related behaviors [9]. The oral health of children is associated with several determinants, including the level of oral health literacy among caregivers and children, adherence to appropriate dental care practices, and accessibility to dental healthcare services [10,11,12].

Distraction is an effective management strategy for alleviating the manifestations of diseases and interventions, including pain and anxiety. Studies have shown that VR distraction significantly reduces pain perception and state of anxiety in children aged five to eight years during short invasive dental treatments [13,14]. A systematic review conducted by Goettems et al. in 2017 analyzed various non-pharmacological methods for distracting children's attention and relieving their pain and anxiety during dental treatments, finding mixed but generally positive impacts on anxiety and pain perception [15]. The distracting function of VR is mediated by reducing sensory signals through frontal cortical processes, highlighting its reliance on the neurobiological condition of the brain [16]. This underscores that neuropsychological health and the presence of concurrent medical conditions in individuals also exert a substantial influence on the outcomes of utilizing such technological interventions.

The current study provides valuable insights into the advantages of employing VR devices in mitigating children's anxiety while visiting dental clinics. By comparing values of some vital measurements such as the SEM scale, pulse rate, SpO_2_ values, and behavior scales, we could assess the effectiveness of the VR devices on the perceptions and attitudes of children towards dental treatment. Similar to our study, parameters such as SEM (sensation-eye-movement), pulse rate, and SpO_2_ levels have been used as reference measures to assess the effectiveness of various intervening items in managing pain during dental procedures [17,18]. Additionally, the utilization of behavior assessment tools such as FBRS and VPT has been observed in various research studies aimed at examining dental anxiety and assessing OHRQOL among children undergoing dental interventions using different pain-relieving techniques. These tools provide valuable insights into the behavioral and psychological aspects of pediatric dental care [19-20]. Based on our findings, there were no considerable differences in the vital metrics recorded before and after the utilization of VR glasses. This suggests that the practicality of using such glasses may be limited.

However, disparities in findings across various reports investigating the impact of VR on children during dental treatment emphasize the presence of additional underlying contributing factors that influence their responses. Studies involving older children aged 5-12 years undergoing dental treatment with VR showed reduced levels of pain and anxiety [2]. Atzori et al. (2018) provided evidence supporting the efficacy of VR as an analgesic technology during dental procedures, particularly for older children [21]. These findings suggest that age and developmental stage significantly influence cognitive abilities and adaptive strategies toward innovative interventions. The number of dental visits and the type of dental procedure also determine children's cognitive perception of such events [22]. Another crucial determinant is the quality and content of VR devices, which are pivotal in defining their effectiveness. Interestingly, game-based VR systems have demonstrated the highest effectiveness and positive outcomes among children during medical interventions. This is attributed to their ability to fully engage and interact with games, making them especially valuable for distraction from pain or anxiety [23].

The role of dentists in providing behavior guidance is pivotal for ensuring the safe and comfortable delivery of dental care, significantly shaping the perceptions of children and their parents regarding any treatment [24]. Furthermore, prior experiences in the dental examination setting, whether positive or negative, substantially impact a child's attitude toward visiting the dentist [25]. Consequently, negative experiences can play a crucial role in creating varying degrees of pain and anxiety in children, affecting their willingness to seek dental care in the future.

Addressing ethical considerations is crucial when using VR in dental care, particularly in obtaining informed consent and ensuring age-appropriate content for children. Recent studies emphasize the need for VR to be used effectively and sensitively, tailored to the unique needs of pediatric patients [26,27]. This approach ensures the responsible and ethical use of VR in pediatric dentistry.

Limitations

This study has a few limitations, which highlight the need for longitudinal research to assess the long-term effects of VR use in pediatric dentistry. The small sample size and the study's focus on a limited range of settings restrict the generalizability of the findings. Future research should involve diverse cohorts, including children from various backgrounds, and be conducted in multiple dental care environments to better understand the broader impact of VR. Additionally, incorporating qualitative methods could provide deeper insights into the subjective experiences of children and their parents with VR in dental settings.

## Conclusions

Our findings reject the assumption that the utilization of VR devices could help to distract children’s attention and reduce their anxiety and pain during dental procedures. This is substantiated by the observation that all vital parameters remained nearly similar when recorded before and after the dental interventions, irrespective of whether VR devices were employed or not. Other effective cognitive-based management techniques should be experimented with to relieve the physical and psychological burden on children during any dental treatment sessions. Furthermore, collaborative efforts between both caregivers and dentists, aimed at tailoring novel stress-relief approaches for children before treatment interventions, could lead to more favorable results.
